# Prophylactic and Therapeutic Effects of Oleuropein on Reperfusion-Induced Arrhythmia in Anesthetized Rat

**DOI:** 10.7508/ibj.2016.01.006

**Published:** 2016-01

**Authors:** Babak Baharvand, Mansour Esmailidehaj, Jamileh Alihosaini, Shirin Bajoovand, Saeedeh Esmailidehaj, Zeynab Hafizibarjin

**Affiliations:** 1 Heart Center, Lorestan University of Medical Sciences, Khoramabad, Iran;; 2 Dept. of Physiology, Faculty of Medicine, Shahid Sadoughi University of Medical Sciences, Yazd, Iran;; 3 Faculty of Medicine, Shahid Sadoughi University of Medical Sciences, Yazd, Iran

**Keywords:** Oleuropein, Arrhythmia, Reperfusion, Rats

## Abstract

**Background:**

This study was conducted to reveal that whether i.v. injection of oleuropein, the most potent polyphenolic antioxidant in olive leaf, has any effect on the magnitude of reperfusion arrhythmia in anesthetized rats or not.

**Methods:**

Eighty male Wistar rats were divided into 8 groups of 10 each: groups 1 and 5 were assigned as the prophylactic and treatment control groups, groups 2 and 6 as the prophylactic and treatment groups with lidocaine (10 mg/kg), groups 3 and 4 as the prophylactic groups with 10 and 50 mg/kg oleuropein (i.v.), and groups 7 and 8 as the treatment groups with 10 and 50 mg/kg oleuropein (i.v.), respectively. Reperfusion injury was induced by 5-min regional ischemia and 15-min reperfusion of left anterior descending coronary artery. Heart rate, blood pressure, and electrocardiogram were monitored throughout the procedure.

**Results:**

blood pressure was significantly decreased by infusion of 50 mg/kg oleuropein in groups 4 and 8, but unlike the lidocaine as a standard anti-arrhythmic drug in groups 2 and 5 had not significant effect on heart rate. The onset of arrhythmia in groups received oleuropein (groups 3, 4, 7, and 8) was significantly delayed. The mortality rate due to irreversible ventricular fibrillation was also significantly reduced in groups 3, 4, 7, and 8. The effect of lidocaine in groups 2 and 5 was more potent than that in oleuropein group.

**Conclusion:**

These findings indicate that i.v. injection of oleuropein possibly through its antioxidant activity reduces the magnitude of reperfusion-induced arrhythmia.

## INTRODUCTION

Although restoration of blood flow to the ischemic myocardium is an important prerequisite for its survival, there is a lot of evidence that reperfusion, apart from its beneficial effects, causes adverse effects known as "reperfusion injury"^[^^1^^,^^2^^]^. Reperfusion injury includes myocardial necrosis, incidence of life-threatening arrhythmia, stunning, and endothelial dysfunction^[^^3^^]^. This damage is mainly attributed to the overproduction of free radicals such as superoxide anions, hydroxyl radicals, hydrogen peroxide, and peroxynitrite within the cell^[^^4^^]^. Thus, it seems that increasing the antioxidant defense of tissues before restoration of blood flow could prevent reperfusion injury. Some previous studies have shown that the consumption of antioxidants such as superoxide dismutase, N-acetylcysteine, allo-purinol, vitamins E and C, and glutathione reduces the reperfusion injury^[^^5^^,^^6^^]^. In contrast, some other studies have indicated that antioxidants in experimental and clinical settings are unable to create protection against this injury^[^^3^^,^^7^^-^^9^^]^. Perhaps, it is due to the possibility of multiple mechanisms involved in reperfusion injury. Therefore, the use of compounds, which can inhibit most of the mechanisms involved in reperfusion, may be more effective in the prevention and treatment of reperfusion injury. 

Today, biologically active substances, especially antioxidants with plant origin have been devoted to the main branch of modern medical therapy. One of these powerful polyphenolic antioxidants is oleuropein. Oleuropein is a phenolic compound from secoiridoids family isolated from olive leaves almost a century ago^[^^10^^]^. Oleuropein exists in all parts of the olive tree, including root, stem, leaf, and fruit^[^^11^^]^. Oleuropein content of olive leaf is about 1-14% of its dry weight^[^^12^^]^. This compound has a powerful antioxidant activity and is a free radical scavenger in *in vivo* and *in vitro* conditions^[^^12^^-^^14^^]^. These biological activities of oleuropein are comparable to Vitamin E^[^^15^^]^.

Many studies have indicated that oleuropein in addition to its antioxidant activity has several other biological benefits, including spasmolytic^[^^13^^]^, anti-inflammatory^[^^12^^,^^16^^]^, hypotensive^[^^17^^]^, anti-infarct^[^^14^^]^, cardio-protective^[18]^, endothelial cell protective^[^^19^^]^, anti-platelet^[^^20^^,^^21^^]^, immunomodulator^22^, and anti-microbial^[^^23^^]^ activities.

In our previous study, we observed that administration of a single dose of oleuropein (100 mg/kg, intraperitoneally) before removing the heart reduced the severity of injury caused by ischemia-reperfusion in isolated rat heart^[^^24^^]^. We also observed that oral administration of oleuropein (20 mg/kg) for at least four weeks can reduce the magnitude of aconitine-induced arrhythmia^[^^25^^]^. In 1978, Petkov and Manolov^[^^17^^]^ reported that oleuropein can prevent calcium chloride-induced arrhythmia and increase the lifetime of animals after the infusion of aconitine in rats, but has not any effect on barium chloride-induced arrhythmia in rabbits, strophanthin-induced arrhythmia in cats and adrenaline-induced arrhythmia in rats.

The main purpose of this study was to investigate the prophylactic and therapeutic effects of i.v. administration of oleuropein on reperfusion-induced arrhythmia in anesthetized rats and compare those with lidocaine as a standard anti-arrhythmic drug.

## MATERIALS AND METHODS

**Animals**

To perform this study, male Wistar rats weighing 250-350 g were used. The animals were housed in polyethylene cages in a humid room (55%) with 22 ± 2^o^C and 12-hour light/dark cycles. All surgical procedures were approved by the Animal Care and Use Committee of Shahid Sadoughi University of Medical Sciences, Yazd, Iran. 

**Experimental grouping**

In total, 80 male Wistar rats were divided into 8 groups of 10 in each. Groups 1-4 were considered as the prophylactic groups and groups 5-8 as the treatment groups as follows:

Group 1 as the prophylactic sham group (Sham-p group): rats were given 1 ml normal saline (i.v.) as a vehicle two minutes before ischemia; Group 2 as the prophylaxis with lidocaine (Lido-p group): rats were given 10 mg/kg lidocaine in 1 ml normal saline (i.v.) two minutes before ischemia (as the positive control group); Group 3 as the prophylaxis with 10 mg/kg oleuropein (Ole10-p group): rats were given 10 mg/kg oleuropein in 1 ml normal saline (i.v.) two minutes before ischemia; Group 4 as the prophylaxis with 50 mg/kg oleuropein (Ole50-p group): rats were given 50 mg/kg oleuropein in 1 ml normal saline (i.v.) two minutes before ischemia; Group 5 as the treatment sham group (Sham-t group): rats were given 1 ml normal saline (i.v.) two minutes before reperfusion; Group 6 as the treatment with lidocaine (Lido-t group): rats were given 10 mg/kg lidocaine in 1 ml normal saline (i.v.) two minutes before reperfusion (as the positive control group); Group 7 as the treatment with 10 mg/kg oleuropein (Ole10-t group): rats were given 10 mg/kg oleuropein in 1 ml normal saline (i.v.) two minutes before reperfusion; Group 8 as the treatment group with 50 mg/kg oleuropein (Ole50-t group): rats were given 50 mg/kg oleuropein in 1 ml normal saline (i.v.) two minutes before reperfusion. The above doses were selected based on Petkov and Manolov's study^[^^17^^]^.

**Experimental procedure**

All animals were anesthetized with intraperitoneal injection of 75 mg/kg sodium thiopental (Rotexmedica, Trittau, Germany). Following cannulation of tail vein with an angiocatheter (gauge 23) to inject normal saline, lidocaine (Iran Daru, Iran), or oleuropein (Indofine, Hillsborough, NJ, USA), rats were fixed on a surgical table, and the temperature of their body was maintained between 36.5 and 37.5ºC using a heating pad. Then carotid artery was cannulated to measure arterial blood pressure using the Powerlab Data acquisition system (ADI, Australia). To monitor the electrical activity of the heart and cardiac arrhythmia, lead II of electrocardiogram was used by connecting two electrodes to the right arm and the left leg of the rats. Next, tracheotomy was performed, and animals were artificially ventilated by a small animal ventilator (Harvard, USA) at stroke of 80/min, tidal volume of 1 ml/100 g body weight and 95% oxygen. Thereafter, the chest in the fifth intercostal space was opened, and a 5-0 silk suture was placed around the left anterior descending coronary artery (LAD). After 20 minutes of stabilization, to induce ischemia, the LAD was occluded for 5 min, which was confirmed by ST segment elevation, cyanosis, and hypotension. Finally, LAD was reopened to reperfuse the ischemic myocardium for 15 min, which was confirmed by relieving the cyanosis and ECG changes such as the incidence of life-threatening arrhythmia.

**Fig. 1:**
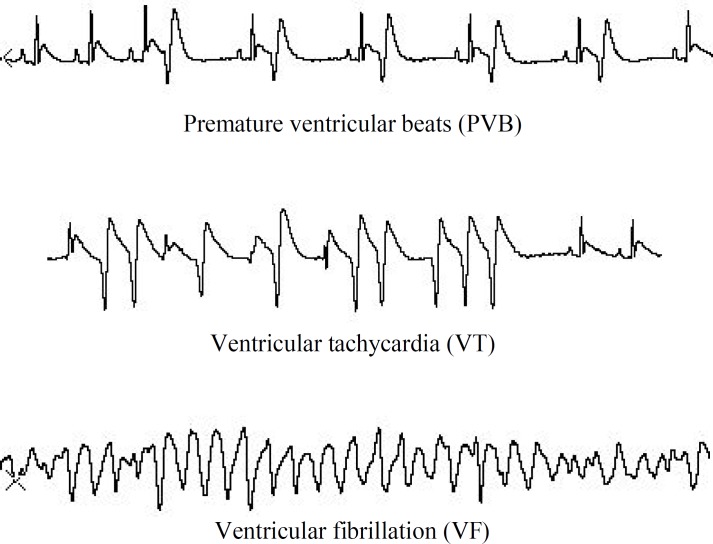
Types of arrhythmia

**Classification of arrhythmia**

Arrhythmias were classified according to the Lambeth conventions^[^^26^^]^: premature ventricular beat (PVB) was considered as a prolonged QRS complex with ventricular origin, more than three consecutive PVBs were considered as ventricular tachycardia (VT), and if there was not a discernible QRS it was considered as ventricular fibrillation (VF). If VF lasted more than 120 minutes, it was considered as an irreversible fibrillation (IVF) or mortality (Fig. 1).

**Exclusion criteria**

Exclusion criteria included no elevation in ST segment of ECG, dysrhythmia, and mean blood pressure below 60 mmHg before the occlusion of LAD and severe atrioventricular block during 5-min ischemia.

**Statistical analysis**

Data were analyzed using Graphpad Prism 5 (San Diego, CA, USA) and showed mean ± SEM and the percentage of incidence. Two-way analysis of variance (two-way ANOVA) was used to evaluate the differences in heart rate, mean arterial pressure, and rate pressure product. For evaluation of differences in arrhythmia parameter, one-way analysis of variance (one-way ANOVA) with Turkey's test as the Post hoc test was used. Fisher's exact test was also used to analyze the percentage of incidence. Finally *P*<0.05 was considered statistically significant.

## RESULTS

**Effects of prophylaxis with lidocaine and oleuropein on heart rate, mean arterial blood pressure, and rate pressure product in rats**

**Fig. 2:**
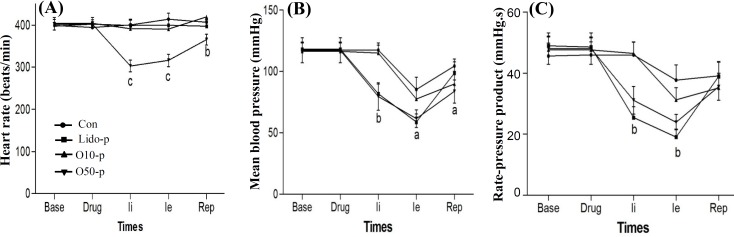
Prophylaxis with oleuropein and lidocaine on heart rate (A), mean blood pressure (B) and rate pressure product (C) before, during and after ischemia. Drug means the infusion of oleuropein or lidocaine; Ii, initiation time of ischemia; Ie, end time of ischemia and Rep, reperfusion time. Con, control group; Lido-p, prophylaxis group with 10 mg/kg lidocaine; Ole10-p, prophylaxis group with 10 mg/kg oleuropein; Ole50-p, prophylaxis group with 50 mg/kg oleuropein. a, *P*<0.05; b, *P*<0.001; c, *P*<0.001 vs. control group

Figure 2A shows that oleuropein (10 and 50 mg/kg) as a prophylaxis caused no significant changes in the heart rate compared to the control group, but lidocaine (10 mg/kg) as a standard anti-arrhythmic drug significantly decreased the heart rate before and during ischemia and reperfusion. Figure 2B indicates the effects of oleuropein (10 and 50 mg/kg) and lidocaine (10 mg/kg), as prophylaxis, on mean arterial blood pressure. Oleuropein at the dose of 10 mg/kg caused no significant change in mean arterial blood pressure compared to the control group, but at the dose of 50 mg/kg significantly reduced it before and during ischemia and reperfusion. Lidocaine (10 mg/kg) significantly reduced mean arterial blood pressure before and during ischemia, but returned near baseline value during reperfusion. Figure 2C demonstrates the effects of oleuropein (10 and 50 mg/kg) and lidocaine (10 mg/kg), as prophylaxis, on rate pressure product (systolic pressure multiple by heart rate). Oleuropein, at the dose of 50 mg/kg, significantly reduced rate pressure product before and during ischemia and returned near baseline value during reperfusion.

**Table 1:** Effects of prophylaxis with lidocaine and oleuropein on the magnitude of reperfusion arrhythmia

**Arrhythmia parameters**	**Con**	**Lido-p**	**Ole10-p**	**Ole50-p**
Onset of arrhythmia (s)	2.6 0.7	7.2 2.3*	6.8 0.9**	9.1 1.3**
Duration of arrhythmia (s)	172.0 41.0	11.7 4.6***	103.0 22.0**	185 23
PVB numbers	52.0 12.0	7.8 2.8**	48.0 10.0	75 13
VT numbers	14.0 1.5	00.0 0.0***	14.0 4.2	23 3.4
VF numbers	3.3 1.0	00.0 0.0***	4.0 1.2	3.9 0.7
VT duration	29.0 4.1	00.0 0.0***	16.0 5.5	26 5.7
VF duration	76.0 28.0	00.0 0.0***	26.0 10.0*	64 21
VT (%)	84	0**	90	80
VF (%)	92	0**	80	100
Mortality rate (%)	25	0**	10***	1***

Data were indicated as mean ± SEM. PVB, premature ventricular beats; VT, ventricular tachycardia, VF, ventricular fibrillation; Con, control group; Ole10-p, prophylaxis group with 10mg/kg oleuropein, O50-p, prophylaxis group with 50 mg/kg oleuropein, Lido-p, prophylaxis group with 10mg/kg lidocaine.

* *P*<0.05;

** *P*<0.01 and

*** *P*<0.001 vs. control group.

**Effects of prophylaxis with lidocaine and oleuropein on the magnitude of reperfusion arrhythmia in rats**

As shown in Table 1, the initiation time of arrhythmia was significantly delayed in Lido-p, Ole10-p, and Ole50-p groups compared to control group. Duration of arrhythmia during reperfusion was markedly reduced in Lido-p and Ole50-p groups. Prophylaxis with 10 and 50 mg/kg oleuropein (Ole10-p and Ole50-p groups) did not have any significant effect on the episodes of PVB, VT, and VF and the incidence and duration of VT and VF during reperfusion, whereas lidocaine markedly reduced them. However, mortality rate due to IVF and severe bradycardia was significantly decreased in all prophylaxis groups. 

**Effects of treatment with lidocaine and oleuropein on heart rate, mean arterial blood pressure, and rate pressure product in rats**

**Fig. 3:**
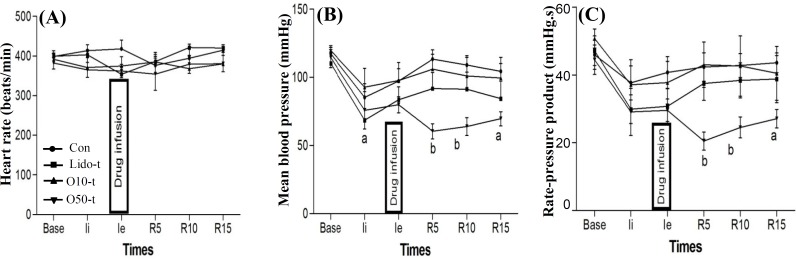
Effects of treatment with oleuropein and lidocaine on heart rate (A), mean blood pressure (B) and rate pressure product (C) before and during reperfusion. Ii, initiation time of ischemia; Ie, end time of ischemia; R5, R10, and R15, 5, 10, and 15 minutes after reperfusion, respectively. Con, control group; Lido-t, treatment group with 10 mg/kg lidocaine; Ole10-t, treatment group with 10 mg/kg oleuropein; O50-t, treatment group with 50 mg/kg oleuropein. a, *P*<0.05; b, *P*<0.01 vs. control group

Three minutes before reperfusion of ischemic myocardium, lidocaine and oleuropein were injected into the blood to diffuse throughout the blood except into the ischemic area. As Figure 3A shows, lidocaine and oleuropein did not have any significant effect on heart rate before and during reperfusion. Treatment with lidocaine and oleuropein 10 mg/kg did not have any significant effect on mean blood pressure (Fig. 3B) and rate pressure product (Fig. 3C) during reperfusion, whereas treatment with oleuropein 50 mg/kg significantly reduced the mean blood pressure and rate pressure product during reperfusion (Fig. 3C).

**Effects of treatment with lidocaine and oleuropein on the magnitude of reperfusion arrhythmia in rats**

Table 2 shows that the initiation time of arrhythmia was significantly delayed only in Lido-t and Ole50-t groups. Duration of arrhythmia and the episodes of PVB were not significantly different between Ole10-t and Ole50-t groups and control group, whereas they were considerably reduced in Lido-t group compared to control group. The incidence of VT was markedly reduced in lido-t and Ole10-t groups, but there was not any significant difference between Ole50-t group and control group. The incidence of VF and IVF was significantly reduced only in Lido-t group and Ole50-t groups, respectively. Although the number of VF was not significantly different among all the groups, the duration of VF was significantly reduced in all treatment groups. 

## DISCUSSION

The main findings of this study show that i.v. injection of a single dose of oleuropein (10 and 50 mg/kg) prior to coronary artery ligation (as prophylaxis) or before and during reperfusion (as treatment) has anti-arrhythmic effects against reperfusion-induced arrhythmia that was evident with delayed initiation of arrhythmia and decreased incidence of IVF (or mortality). The anti-arrhythmic effects of oleuropein was not as potent as lidocaine as a positive control, anti-arrhythmic drug in clinic. On the other hand, in contrast to lidocaine that reduced heart rate, oleuropein did not have any significant effect on heart rate as prophylaxis and treatment. However, oleuropein 50 mg/kg reduced mean arterial pressure and rate pressure product significantly. 

Ventricular arrhythmia is the most important complication following reperfusion of ischemic myocardium. This kind of arrhythmia is observed in conditions such as coronary spasm, angioplasty or thrombolysis and cardiac surgery with ischemic arrest^[^^14^^]^. Many factors affect the onset of arrhythmia, but defect in the antioxidant defense system of ischemic myocardium is the main factor^[3]^. Generation of reactive oxygen species such as superoxide anions, hydroxyl radicals, and hydrogen peroxide especially in early phases of reperfusion contribute to the incidence of arrhythmia^[^^27^^]^. Reperfusion arrhythmia can be suppressed by antioxidant enzymes, free radical scavengers, and inhibitors of free radical production^[^^13^^]^. Therefore, the potentiation of the myocardial antioxidant system will reduce the incidence of arrhythmia^[^^28^^]^. The purpose of this study was to know whether antioxidant activity of oleuropein could protect heart against reperfusion-induced arrhythmia in anesthetized rat or not.

Oleuropein is a phenolic compound that was first isolated from olive leaf about a decade ago^[^^10^^,^^29^^]^. Oleuropein is the dominant compound of olive leaf extracts on the market, and it has been stated that the biological benefits of olive leaf extracts are positively correlated with its content of oleuropein^[^^13^^]^. Several studies have reported the beneficial effects of oleuropein including anti-diabetic^[^^30^^]^, anti-inflamm-atory^[^^31^^]^, anti-apoptotic^[^^15^^]^, anti-infarct^[^^13^^]^, and anti-microbial^[^^32^^]^ effcets, and protection of the skin against ultraviolet ray^[^^33^^,^^34^^] ^etc. It seems that most of the biological effects of oleuropein are related to its antioxidant properties. Antioxidant activity of oleuropein is related to its catechol group, which stabilizes free radicals through the formation of intermolecular hydrogen bonds^[^^13^^]^.

**Table 2:** Effects of treatment with lidocaine and oleuropein on the magnitude of reperfusion arrhythmia

**Arrhythmia parameters**	**Con**	**Lido-t**	**Ole10-t**
Onset of arrhythmia (s)	2.6 0.7	10.6 1.8*	4.6 1.2
Duration of arrhythmia (s)	172.0 41.0	72.0 25.0**	15.0 98.0
PVB numbers	52.0 12.0	17.0 4.1**	59.0 27.0
VT numbers	14.0 1.5	4.0 1.1**	18.0 2.5
VF numbers	3.3 1.0	3.0 1.2	4.5 1.3
VT duration	29.0 4.1	16.0 6.0	16.0 4.0
VF duration	86.0 30.0	6.5 0.5**	12.0 3.1**
VT (%)	84	50**	40***
VF (%)	92	50**	100
Mortality rate (%)	25	20	50

PVB, premature ventricular beats; VT, ventricular tachycardia, VF, ventricular fibrillation; Con, control group; Ole10-t, treatment group with 10mg/kg oleuropein, O50-t, treatment group with 50mg/kg oleuropein, Lido-t, treatment group with 10mg/kg lidocaine.

* *P*< 0.05;

** *P*<0.01 and

*** *P*<0.001 vs. control group.

There are few studies on oleuropein and its effects on cardiovascular hemodynamics and cardiac arrhythmia. For the first time in 1978, Petkov and Manolov^[^^17^^]^ reported the anti-arrhythmic effects of oleuropein. They revealed that oleuropein disappeared calcium chloride-induced arrhythmia in rats and increased animal lifetime against aconitine-induced arrhythmia, whereas it did not have beneficial effect on arrhythmia induced by barium chloride in rabbits, by adrenaline in rats and by strophanthin in cats. Our previous study showed that injection of a single dose of intraperitoneal oleuropein (100 mg/kg) had anti-arrhythmic effects against ischemic-reperfusion injury in isolated rat heart^[^^24^^]^. In our another study, we observed that long-term consumption of oleuropein (20 mg/kg/day for at least four weeks) reduced the severity of aconitine-induced arrhythmia^[^^25^^]^. In the present study, we used a single dose of two different doses of oleuropein (10 and 50 mg/kg, i.v.) as prophylaxis and treatment, respectively. It had hypotensive and anti-arrhythmic effects especially at dose of 50 mg/kg. We also used lidocaine as a positive control that is used commonly as a standard anti-arrhythmic drug in clinic^[^^13^^]^. The anti-arrhythmic action of oleuropein was considerably weaker than that of lidocaine. These data are approximately in line with those of Petkov and Manolov's study^[^^17^^]^ with the exception that they used only one dose of oleuropein (40 mg/kg, i.v.) as prophylaxis.

In 2004, Manna and colleagues^[^^18^^] ^used isolated rat heart perfused with an oleuropein solution. They reported that oleuropein had a cardioprotective effect against ischemic-reperfusion injury that was associated with decreased level of creatine kinase and malondialdehyde and increased level of antioxidant in coronary effluent. However, they did not mention the incidence of arrhythmia and heart function. In 2007, Andreadou *et al.*^[^^14^^]^ demonstrated that oral administration of 10 and 20 mg/kg oleuropein for 3 to 6 weeks to rabbits receiving a high cholesterol diet had anti-arrhythmic, antioxidant and hypoglycemic effect. They also indicated that intraperitoneal injection of oleuropein (100 and 200 mg/kg) had cardioprotective effect against doxorubicin cardiotoxicity through modification of metabonomic activity of the heart in rat^[^^13^^]^.

In the present study, Although the mechanisms involved in the protective effects of oleuropein was not studied, it seems that several mechanisms might contribute to reduce the severity of reperfusion-induced arrhythmia, including reduced oxidative stress^[^^13^^]^, inhibition of platelet aggregation^[^^20^^,^^21^^]^, protection of vascular endothelial cells^[^^14^^]^, inhibition of calcium channels^[^^35^^]^ etc. Therefore, further studies are needed to be performed on the mechanisms involved in the cardiac protection induced by oleuropein.

Another finding of this study was the effect of oleuropein on mean arterial blood pressure, heart rate, and rate-pressure product. Oleuropein had no effect on heart rate in the present investigation; however, it significantly reduced mean arterial blood pressure and rate pressure product at dose of 50 mg/kg. Petkov and Manolov^[^^17^^] ^also reported that i.v. injection of a single dose of oleuropein caused hypotension in conscious cats and dogs. Also, oleuropein increased the coronary blood flow in isolated rat heart by 50%. Oi-Kano et al in 2008 reported that i.v. administration of oleuropein to rats increased plasma levels of adrenaline and noradrenaline^[^^36^^]^. Since the effect of catecholamines on blood pressure and heart rate is dependent on their plasma concentration, Oi-Kano and colleagues^[^^36^^]^ did not report any information about heart rate and blood pressure changes in their study. 

According to the results of the present study, it appears that oleuropein causes hypotension through its vasodilatory effect, because it has not any effect on heart rate. Therefore, further studies are needed to determine the hypotensive mechanisms of oleuropein in the future. On the other hand, we could not find any study on arrhythmia and oleuropein; thus, more research is needed to be carried out in this area.

In the current investigation, the reduced rate pressure product in groups received 50 mg/kg oleuropein indicated that oleuropein may lower the myocardial oxygen consumption which in turn can lead to the reduced incidence of irreversible ventricular fibrillation. The results of this study show that the i.v. administration of oleuropein has anti-arrhythmic and hypotensive effects, especially at dose of 50 mg/kg. Hence, the use of oleuropein and plant extracts containing oleuropein might open a promising window to prevent damages caused by ischemia-reperfusion injuries in cardiovascular and non-cardiovascular surgeries.
